# Ketamine effects on default mode network activity and vigilance: A randomized, placebo‐controlled crossover simultaneous fMRI/EEG study

**DOI:** 10.1002/hbm.24791

**Published:** 2019-09-18

**Authors:** Norman Zacharias, Francesco Musso, Felix Müller, Florian Lammers, Andreas Saleh, Markus London, Peter de Boer, Georg Winterer

**Affiliations:** ^1^ Clinical Neuroscience Research Group, Experimental and Clinical Research Center (ECRC), Department of Anesthesiology and Operative Intensive Care Medicine (CCM, CVK) Charité – Universitätsmedizin Berlin, Humboldt‐Universität zu Berlin, and Berlin Institute of Health Berlin Germany; ^2^ Pharmaimage Biomarker Solutions GmbH Berlin Germany; ^3^ Pharmaimage Biomarker Solutions, Inc. Boston Massachusetts; ^4^ Department of Psychiatry Heinrich‐Heine University Düsseldorf Germany; ^5^ Institut für Diagnostische und Interventionelle Radiologie und Kinderradiologie Klinikum Schwabing Munich Germany; ^6^ Early Development and Clinical Pharmacology Janssen‐Cilag GmbH Neuss Germany; ^7^ Janssen Pharmaceutica Johnson & Johnson Pharmaceutical Research and Development Beerse Belgium

**Keywords:** antidepressant action, depression, EEG‐fMRI, ketamine, vigilance control

## Abstract

In resting‐state functional connectivity experiments, a steady state (of consciousness) is commonly supposed. However, recent research has shown that the resting state is a rather dynamic than a steady state. In particular, changes of vigilance appear to play a prominent role. Accordingly, it is critical to assess the state of vigilance when conducting pharmacodynamic studies with resting‐state functional magnetic resonance imaging (fMRI) using drugs that are known to affect vigilance such as (subanesthetic) ketamine. In this study, we sought to clarify whether the previously described ketamine‐induced prefrontal decrease of functional connectivity is related to diminished vigilance as assessed by electroencephalography (EEG). We conducted a randomized, double‐blind, placebo‐controlled crossover study with subanesthetic S‐Ketamine in *N* = 24 healthy, young subjects by simultaneous acquisition of resting‐state fMRI and EEG data. We conducted seed‐based default mode network functional connectivity and EEG power spectrum analyses. After ketamine administration, decreased functional connectivity was found in medial prefrontal cortex whereas increased connectivities were observed in intraparietal cortices. In EEG, a shift of energy to slow (delta, theta) and fast (gamma) wave frequencies was seen in the ketamine condition. Frontal connectivity is negatively related to EEG gamma and theta activity while a positive relationship is found for parietal connectivity and EEG delta power. Our results suggest a direct relationship between ketamine‐induced functional connectivity changes and the concomitant decrease of vigilance in EEG. The observed functional changes after ketamine administration may serve as surrogate end points and provide a neurophysiological framework, for example, for the antidepressant action of ketamine (trial name: 29JN1556, EudraCT Number: 2009‐012399‐28).

## 1 INTRODUCTION

The default mode network (DMN) during resting‐state (rs) condition constitutes a network of brain regions, including the medial prefrontal cortex (mPFC), posterior cingulate cortex (PCC)/precuneus, medial, lateral, and inferior parietal cortex. These regions become simultaneously active when subjects are self‐referential and not focused on the outside world with the brain being at wakeful rest (Buckner, Andrews‐Hanna, & Schacter, 2008; Lemogne et al., 2010; Lemogne, Delaveau, Freton, Guionnet, & Fossati, 2012). Depressed patients tend to be self‐referential (Bergouignan et al., 2008; Grimm et al., 2009) and several studies have consistently demonstrated increased DMN functional connectivity in depressed patients (Greicius et al., 2007; Sheline et al., 2009; Sheline, Price, Yan, & Mintun, 2010; Zhu et al., 2012). Accordingly, DMN functional activity is of particular interest as a potential mechanistic marker of depression and antidepressant treatment‐response. Using resting‐state fMRI (rsfMRI) measures such as functional connectivity in DMN as a mechanistic and treatment response marker, however, may come with a major drawback. As recently pointed out by Tagliazucchi and Laufs (Tagliazucchi & Laufs, 2014) in a large multicentric study of 1,147 rsfMRIs from the “1000 Functional Connectomes Project”, resting state is an uncontrolled condition and its heterogeneity is neither sufficiently understood nor accounted for. Based on a long‐standing tradition in electroencephalography (EEG) research to use EEG as an objective measure of vigilance (Ott, McDonald, Fichte, & Herrmann, 1982) and using simultaneously acquired EEG during rsfMRI measurements, one‐third of subjects were found to exhibit unstable wakefulness and a loss of wakefulness within 3 min. In their study, these dynamic changes of wakefulness were associated with fundamental changes in the associated BOLD responses (functional connectivity). The authors concluded from their findings that vigilance or vigilance monitoring is required when using rsfMRI as a functional biomarker. For obvious reasons, this kind of monitoring is even more required when drugs are investigated which may affect the level of vigilance and consciousness such as the anesthetic ketamine or related drugs. For instance, Bonhomme et al. (2016) recently reported a breakdown of DMN functional connectivity between PFC and PCC during stepwise increase of ketamine (or propofol) dosage when comparing the level of sedation during wake state and light and deep sedation (until unresponsiveness). Accordingly, what is needed is an integration of the fMRI findings on the action of ketamine on DMN functional connectivity into a mechanistic neurophysiological model, which accounts for the dynamics of vigilance since even dynamic resting‐state analysis techniques with its temporal resolution in a subsecond scale (Brinkmeyer et al., 2010; Zalesky, Fornito, Cocchi, Gollo, & Breakspear, 2014) might not be sufficient enough. Thus by extension, simultaneous assessment of fMRI data and vigilance using a neurophysiological measure such as EEG may improve our understanding of the antidepressant ketamine action.

McKinnon et al. (2018) recently reported an association of sleep disturbance and DMN functional connectivity in patients with a lifetime history of depression. They found an increased DMN functional connectivity in depressed patients, most notably in depressed patients with concomitant sleep disorder but increased DMN functional connectivity was also seen in nondepressed control subjects with disturbed sleep. In many cases disturbed sleep emerges even before the onset of clinical depression, up to 90% of people with depression complain about diminished sleep quality, which often includes self‐referential, agonizing rumination while being awake during the night (German: “Nächtliches Grübeln”) (Benjamins et al., 2017; Nolen‐Hoeksema, Wisco, & Lyubomirsky, 2008). At the same time, this reduced sleep quality is associated with altered state of vigilance regulation during daytime—in particular hyperarousal (Riemann et al., 2010; Ulke et al., 2017). Also, many antidepressants decrease vigilance (Alberti, Chiesa, Andrisano, & Serretti, 2015; Hensch et al., 2015)—in addition to their effect on frontal functional connectivity (see above)—and it has been suggested that this effect on vigilance contributes to their antidepressant effects (Hegerl & Hensch, 2014). On the basis of these studies, one could expect that the counter‐acting effect of (antidepressant) subanesthetic ketamine on DMN functional connectivity is related to a decrease of the level of vigilance. If this notion were correct, the antidepressant effects of ketamine on functional connectivity and vigilance may constitute two sides of the same coin. In such a scenario, the ketamine effect on vigilance within the framework of resting‐state fMRI study is not a nuisance parameter that needs to be corrected for. Rather, the parallelism of drug effects would (a) provide a more complete picture of the potential antidepressant ketamine effect and (b) improve our understanding of the pathophysiology of depression.

In an earlier study, we have already argued that an effect of subanesthetic doses of ketamine on vigilance is most likely (Musso et al., 2011). Accordingly, the notion that subanesthetic ketamine is decreasing vigilance, similar to light sleep, is consistent with previous resting EEG studies during subanesthetic ketamine challenge (Maksimow et al., 2006; Muthukumaraswamy et al., 2015). These two subanesthetic ketamine EEG studies reported an increase of slow wave power with a concomitant reduction of alpha power—an EEG pattern that is typically seen during light sleep according to the sleep staging of Rechtschaffen and Kales (1968). Similarly, an increase of slow wave activity is also seen during increasing levels of anesthesia (Ching & Brown, 2014; Purdon et al., 2013). However, both EEG studies during subanesthetic ketamine infusion also reported an increase of high‐frequency EEG (gamma) activity which was also found by others but with no concomitant increase of slow wave power (de la Salle et al., 2016; Hong et al., 2010; Lazarewicz et al., 2009). These seemingly discrepant findings (theta vs. gamma activity) are not entirely unexpected. Light sleep or sleep stage 1 (according to Rechtschaffen & Kales, 1968) is a transitory and dynamic stage between wake and sleep, it is an unstable (arousable) sleep stage with relatively frequent so‐called cyclic alternating patterns (CAPs) compared to deep sleep (Terzano et al., 2001). In EEG, CAPs are characterized by transient slow wave oscillations (i.e., decreased vigilance) but also intermittent low amplitude high frequency oscillations up the gamma frequency range, that is, desynchronized EEG vigilance (Parrino, Grassi, & Milioli, 2014; Simor, Gombos, Szakadát, Sándor, & Bódizs, 2016). Interestingly, Ferri, Rundo, Bruni, Terzano, and Stam (2007) could show that the slow wave CAPs possess small world properties with high cluster coefficient and small path length. Accordingly, one could expect that after subanesthetic ketamine administration, an increase of slow frequency theta/delta EEG activity (or an increase of EEG gamma activity) during resting state, which indicates increased small world properties in frontal brain regions (Ferri et al., 2007), would be paralleled by a frontal decrease of DMN functional connectivity to parietal brain regions.

In this study, we sought to clarify whether subanesthetic ketamine effects on DMN connectivity (reduction of connectivity) are related to ketamine effects on vigilance with an increase of slow‐frequency and high‐frequency EEG activity and a concomitant decrease of alpha EEG activity (sleep stage 1). For this purpose, we analyzed data from a randomized, placebo‐controlled crossover study with subanesthetic S‐Ketamine study applying simultaneous rsfMRI/EEG. This is of special interest when having in mind that the Federal Drug Administration (FDA) approved the first ketamine‐based medicine (S‐ketamine nasal spray) for the treatment of (otherwise treatment‐resistant) depression (Carey, 2019).

## 2 METHODS

The study was conducted in compliance with the declaration of Helsinki and in agreement with the Good Clinical Practice (ICH‐GCP) guidelines and the EU Clinical Trial Directive 2001/20/EC (Eudract trial name: 29JN1556, Eudract CT Number: 2009‐012399‐28) and relevant legal regulations of the German Medicines Law (Arzneimittelgesetz). The study was approved by the local ethics committee (Ärztekammer Nordrhein, Düsseldorf, Germany) and by the German federal drug agency (Bundesinstitut für Arzneimittel and Medizinprodukte, BfArM). Written informed consent was obtained from all participants.

### 2.1 Subjects and study design

Inclusion and exclusion criteria of the subjects and the study design were explained in detail in an earlier publication (Musso et al., 2011). In short, during this randomized, double‐blind, placebo‐controlled crossover trial, *N* = 24 healthy, young male subjects without drug treatment at least 4 weeks before study inclusion and with no prior history of a neuropsychiatric disorder were investigated twice at least 1 week apart. Timing between both measurements was chosen to avoid possible residual drug effects on the following crossover scan. As mentioned in our previous publication (Musso et al., 2011), all participants underwent a full examination (medical, neurological, and psychiatric) by a board certified psychiatrist and neurologist. Subjects with a positive history of clinically significant medical/neurological/psychiatric conditions were excluded. Sixteen subjects were nonsmokers, while eight subjects were current smokers. To minimize any smoking effects on vigilance, the study setup followed a standardized operating procedure and the subjects were not allowed to smoke at least 1 hr before start of the resting‐state measurement. Additionally, possible drug use was tested with a urine drug screening for amphetamine, barbiturate, benzodiazepine, cannabinoids, cocaine, and opioids. Subjects with a positive test result were excluded from the study. Study subjects and the involved clinicians and researchers were “blinded.” For both investigations, the subject stayed overnight in a clinical research unit (CRU) of the clinical research organization FOCUS Drug Development GmbH (Neuss, Germany), then underwent the drug‐challenge investigation with simultaneously acquired fMRI/EEG during the following day and leave the CRU after 24 hr under medical supervision. The randomization was carried out prior by the statistical staff of the CRU using block randomization with blocks of size 4.

**Figure 1:**
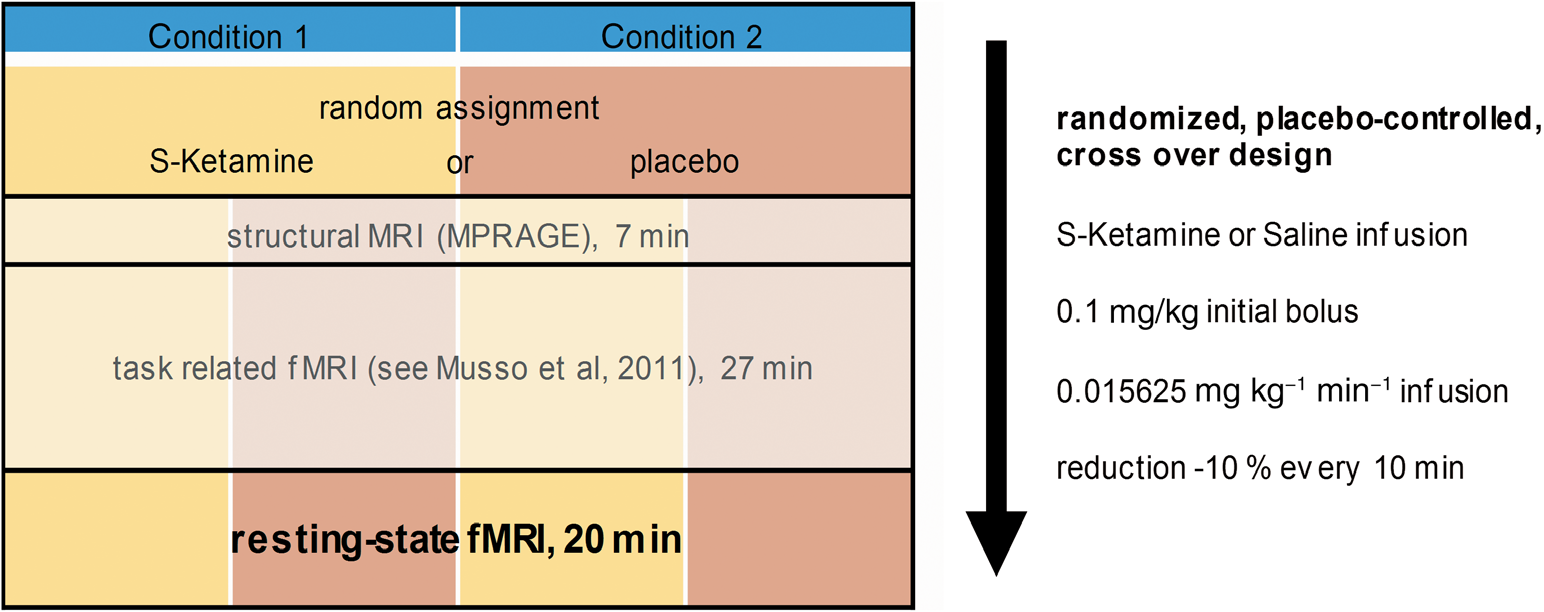
The experimental design including scanning sequences and information about drug application. During the neuroimaging investigation, subjects received in random order either a subanesthetic dose of S‐Ketamine HCl (Esketaminehydrochlorid) [Ketanest® S, Pfizer] or placebo (crossover design, 1 week apart), both administered intravenously in 0.9% NaCl. This administration was carried out as a bolus of S‐Ketamine (0.1 mg/kg during 5 min) immediately before measurements in the MRI scanner and a continuous infusion of S‐Ketamine (0.015625 mg kg^−1^ min^−1^ for the duration of the investigation, i.e., 1 hr maximum) during the measurement. To avoid a slow increase of ketamine plasma levels, a reduction by 10% every 10 min (Umbricht et al., 2000) was determined. The resting‐state fMRI measurement started 34 min after the beginning of the ketamine infusion

For details of drug application and fMRI/EEG study design, see Figure 1. In short, either subanesthetic *S*‐ketamine (*Ketanest® S*, Pfizer Pharma PFE, Berlin, Germany) in 0.9% NaCl or saline (0.9% NaCl) was intravenously administered before and during the MR scan. As of its pharmacokinetics, immediately before starting the MR measurement a bolus of 0.1 mg/kg *S*‐ketamine (or equal volumes of saline) was administered over 5 min. For 1 min after MR scan initiation, the infusion was stopped to reach equilibrium of the ketamine plasma levels. Afterward, the infusion was continued with 0.015625 mg kg^−1^ min^−1^ (max. 1 hr). Since ketamine slowly increases when it is constantly administered (Feng et al., 1995; Umbricht et al., 2000), a dosage reduction of 10%/10 min was used to maintain stable ketamine plasma levels during the experiment. Out of 24 recruited subjects, seven did abort the measurements before the resting‐state measurements could be performed (for sociodemographic details and reasons for exclusion, see Table 1).

**Table 1:** *N* = 17 study subjects with complete data sets

Mean age (median) ± *SD* (years)	27.5 (28.0) ± 4.6
Mean height ± *SD* (cm)	178.2 ± 6.3
Mean weight ± *SD* (kg)	76.8 ± 9.1
Mean BMI ± *SD* (kg/m^2^)	24.2 ± 2.8

*Note*: Out of 24 eligible healthy male volunteers, five subjects were excluded because of incomplete data sets. Furthermore, two subjects did not finish the measurements because of adverse events (one subject with tachycardia, one subject with panic attack). Of the remaining cohort, seven subjects were smokers and 16 subjects were right‐handed.

Abbreviation: BMI, body mass index.

### 2.2 Simultaneous rsfMRI/EEG data acquisition

The MR scans were acquired using a 3T MR Scanner (Trio, Siemens, Erlangen, Germany) at the Heinrich‐Heine University Hospital (Düsseldorf, Germany). The MRI scanning sequence consisted of a structural MR scan (T1‐weighted MR sequence: TR/TE = 2250/3.03 ms, FA = 9°, 176 sagittal slices, FOV = 20 × 20 cm, matrix = 64 × 64, voxel size = 1 × 1 × 1 mm), a visual oddball task (published by Musso et al., 2011) and a resting‐state session (for details of the scanning sequences, see Figure 1). For the resting‐state session, subjects were resting comfortably in the scanner with eyes closed. An echo planar imaging sequence with 42 slices (slice thickness 3 mm) and a 3 mm slice gap was acquired, to cover whole cerebral cortex and cerebellum (TE = 40 ms, TR = 3.4 s, FA = 90°, FOV = 20 × 20 cm, matrix = 64 × 64, voxel size = 3 × 3 × 3 mm). If brain does not fit, lower parts of the cerebellum were cut off during acquisition.

Simultaneously to the functional MR scans, an EEG recording was conducted. An MR compatible EEG setup (Brain Products, Gilching, Germany) was applied including a standard EEG cap (BrainCap MR, EasyCap GmbH, Breitbrunn, Germany) with 32 electrodes (distributed according to 10–20 system, including electrocardiogram attached to the subjects' back and electrooculogram [EOG], attached on the outer canthi of the left eye). EEG data were recorded with a sampling rate of 5 kHz and online bandpass filtered with 0.016–250 Hz. Overall impedances of the recording electrodes were <10 kΩ.

### 2.3 rsfMRI analysis: Seed‐based DMN functional connectivity

For data analysis, the acquired MR data were processed using Matlab‐based CONN connectivity toolbox V17.f (Gabrieli Lab, Massachusetts; Whitfield‐Gabrieli & Nieto‐Castanon, 2012). To reach equilibrium of the spin history, the first five scans of each individual session were discarded. Since three rsfMRI/EEG measurements had to be terminated prematurely because subjects felt uneasy, only data of the first 8.5 min (150 volumes) of the resting‐state session were used for analysis. This is at the lower edge of what has been recently recommended by Birn et al. (2013) as the best length for rsfMRI sessions. It takes into account that a longer duration of resting‐state scanning per se can be associated with functional connectivity fluctuations related to changes in subjects state of vigilance (Tagliazucchi & Laufs, 2014). Preprocessing included the following steps in identical order: realignment, slice‐timing correction, outlier identification via a scrubbing process (using Artifact Detection Tool, ART; Whitfield‐Gabrieli & Nieto‐Castanon, 2012), gray/white matter/cerebrospinal fluid (CSF) segmentation, and normalization of the functional and structural images to Montreal Neurological Institute (MNI) space, as well as coregistration of the individual functional and anatomical images and smoothing (Gaussian kernel of FWHM = 8 mm). To remove confounding sources of signal variation which survived the preprocessing process, the six head motion parameters estimated by the realignment process and the scrubbing covariates were used for denoising the data. Therefore, an anatomical component‐based noise correction method (anatomical CompCor; Behzadi, Restom, Liau, & Liu, 2007) was used, which extracted a representative noise signal from white matter regions and from CSF and removed anything that correlates with those noise components and the aforementioned covariates from the BOLD signal for every voxel. Finally, a band‐pass filter of 0.01–0.1 Hz was applied to the data.

### 2.4 EEG analysis: Preprocessing

EEG preprocessing was done with BrainVision Analyzer 2.1 Professional. The average referenced EEG data were MR and pulse artifact corrected using a sliding average method, down sampled to 500 Hz and filtered (zero phase shift Butterworth filters, 0.53–45 Hz, notch filter of 50 Hz, see Result 1.2 in Supporting Information). In line with rsfMRI processing, EEG data of the first 8.5 min were used for analysis in this article. The continuous EEG data were segmented into 2 s segments with an overlap of 1 s to minimize potential filter artifacts and to achieve a minimal frequency of 0.5 Hz. Since the EOG electrode data were not stable for all subjects, we introduce virtual vertical EOG and horizontal EOG channels by pooling electrode Fp1 and Fp2, or T7 and T8, respectively, and those were used for ocular correction via an ocular artifact‐related subtraction method (Gratton, Coles, & Donchin, 1983) implemented in BrainVision Analyzer 2.1. For further artifact correction, a maximal difference of 210 ± 27 μV for a 1 s data interval was used as threshold. This amounted to 352 ± 106 segments for EEG power analysis.

### 2.5 EEG analysis: Power analysis

On each segment, a fast Fourier transformation (FFT) with a maximum resolution of 0.488 Hz and a periodic Hanning Window of 10% segment length was applied. For frequency band analysis, the resulting power spectra [FFT_P_] were averaged and 11 frequency intervals were defined (delta: 0.53–4 Hz; theta: 4–8 Hz; alpha: 8–12 Hz; alpha1: 8–10 Hz; alpha2: 10–12 Hz; beta: 12–25 Hz; beta1: 12–15 Hz; beta2: 15–18 Hz; beta3: 18–25 Hz; gamma: 30–50 Hz; gamma1: 30–40 Hz). The individual arithmetic mean power of each frequency interval was exported to OriginPro 2017G (^©^OriginLab Corporation, Northampton, MA) for statistical evaluation.

### 2.6 Statistical analyses

This is an exploratory study (placebo‐controlled, randomized, crossover) to understand subanesthetic ketamine effects on brain function—in particular on the relationship between drug‐induced changes of rsfMRI DMN connectivity and vigilance as assessed by EEG power during resting‐state condition with simultaneous rsfMRI/EEG data acquisition. The major hypothesis in this study is that a ketamine‐induced reduction of vigilance (comparable to sleep stage 1) is related to a decrease of frontal rsfMRI DMN connectivity. Coprimary measures are changes of rsfMRI DMN connectivity in the frontal brain region and changes of vigilance using the EEG power spectrum. Secondary measures are the spatial distribution of rsfMRI DMN connectivity in other brain regions, EEG frequency bands, and their spatial distribution across the scalp. For statistical analyses, we performed one‐way repeated measure analysis of variance followed by regression analyses (Pearson's *R*) if not stated otherwise using the OriginPro2017G (^©^OriginLab Corporation) software package. For correlation analysis of EEG versus BOLD activation, a Bonferroni correction was applied due to multiple testing errors. Thus the significance thresholds were *p*_Bonferroni_ = .025 for ΔEEG power versus Δfunctional connectivity analysis (Figure 5c,e) and *p*_Bonferroni_ = .0125 for analysis of drug conditions separately (Figure 5d,f).

### 2.7 Seed‐based functional MRI connectivity analysis

For seed‐based analysis, using the Harvard Oxford Atlas, brain maps of bivariate correlation coefficients were calculated, by correlating the time‐course of the seed region, that is, the filtered data from PCC/precuneus area, a key region of the DMN (Uddin, Kelly, Biswal, Castellanos, & Milham, 2009; Utevsky, Smith, & Huettel, 2014) defined as a sphere of 10 mm radius centered at coordinate 1, −61, 38 (MNI), with time courses of all other brain voxels. The resulting seed‐to‐voxel correlations were entered into a second‐level general linear model. Two‐sided *t* statistics were performed to compare drug condition specific connectivity patterns. To account for multiple comparisons, uncorrected (*p* = .001) voxel‐level height threshold and family‐wise error (FWE) corrected (*p* = .05) cluster‐level extent threshold were used (Whitfield‐Gabrieli & Nieto‐Castanon, 2012). The resulting clusters of significantly different positive or negative connectivity were extracted for further analysis.

### 2.8 EEG power analysis

Due to the fact that electromagnetic data of intrasubject segments did not differ by scaling factors (Zacharias, Sieluzycki, Matysiak, König, & Heil, 2010), individual arithmetic mean power values of each frequency band were imported to the analysis software. Whereas electromagnetic data of different brains are not normally distributed (Gasser, Cher, & Mocks, 1982) and differ by scaling factors (Zacharias, Sieluzycki, Kordecki, König, & Heil, 2011). Therefore, geometric means (GM) over subjects were computed for both conditions and the resulting GM FFT_P_ were plotted as heatmaps on a two‐dimensional surface model of the electrode position. For the same reason, we divided GM FFT_P_ for ketamine condition by GM FFT_P_ for placebo condition and plotted them as heatmap, to get a distribution of scaling factors over the head surface. When analyzing individual data, we account for the non‐normal distribution of FFT_P_ with the use of a logarithmic transformation (ln) (Gasser et al., 1982). For descriptive comparison of ketamine versus placebo, FFT_P_ across electrodes and EEG frequency bands were calculated (*p* = .05, uncorrected).

### 2.9 Relationship between rsfMRI and EEG FFT_P_

We calculated linear regressions (Pearson's *R*) of the DMN functional connectivity against EEG power (logarithmic FFT_P_) to show possible relationships between DMN (rsfMRI) activity and neuronal activation (EEG) both in the placebo and ketamine data sets. We restricted our regression analyses (rsfMRI vs. EEG) to comparable brain regions (e.g., frontal DMN connectivity vs. frontal electrode positions). We performed regression analyses using (a) statistically significant functional connectivities from 2.7 and (b) a corresponding electrode position with the maximum scaling factor of EEG power from 2.8. Besides that, we looked for correlating changes of DMN activity with changes of EEG activity when ketamine was applied. Therefore, the individual differences of ketamine condition minus placebo condition (Δ) (difference values both for EEG power and rsfMRI connectivity) between the two modalities were compared.

## 3 RESULTS

### 3.1 Seed‐based functional connectivity MRI

**Figure 2:**
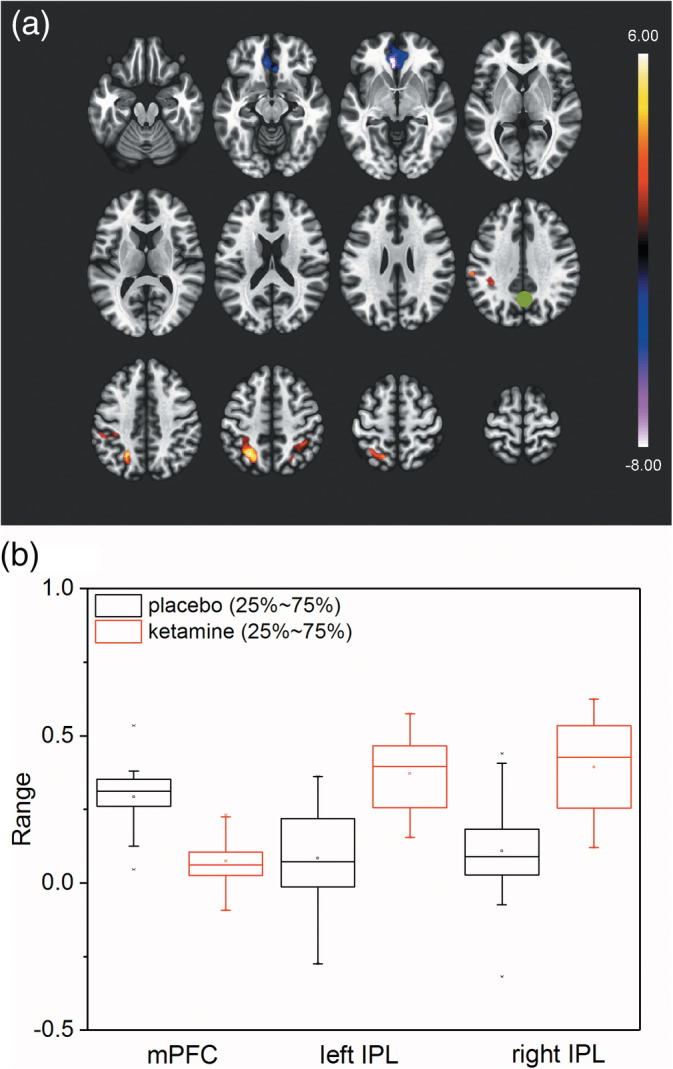
The ketamine effect on seed‐based functional connectivity. (a) Significant *T*‐values (two‐sided) of seed to voxel functional connectivity for ketamine > placebo comparison (FWE cluster‐corrected: *p*_FWE_ = .002). Seed (marked as green dot) is centered at 1, −61, 38 (MNI) and covers PCC/precuneus area. Significant clusters cover mPFC (decreased connectivity) and left and right IPL areas (increased connectivity). (b) Boxplot of individual mean Fisher Z‐transformed functional connectivities for placebo (black) and ketamine (red) condition for the different significant clusters. Of further interest, for ketamine condition, mPFC connectivities were close to zero. Same could be observed for left and right IPL connectivities for placebo condition

The connectivity patterns in the placebo and ketamine condition are shown in the Supporting Information (Result 1.1, Figure S1). Figure 2a shows FWE‐corrected significant connectivity clusters of functional connectivity changes for the contrast ketamine versus placebo. In the mPFC (MNI: −6, 36, −6), we observed decreased connectivity (*p*_FWE_ < .001, *T* = −6.29), whereas in the areas of the left and right intraparietal lobes (IPLs) (MNI: −30, −54, 56 and 34, −46, 50) increased connectivities were found (left IPL: *p*_FWE_ < .001, *T* = 5.49; right IPL: *p*_FWE_ = .001, *T* = 4.87) in the ketamine condition. The resulting individual mean connectivities, separated for the different cluster and conditions, are displayed as boxplots in Figure 2b.

### 3.2 EEG fast Fourier transformation band power (FFT_P_)

**Figure 3:**
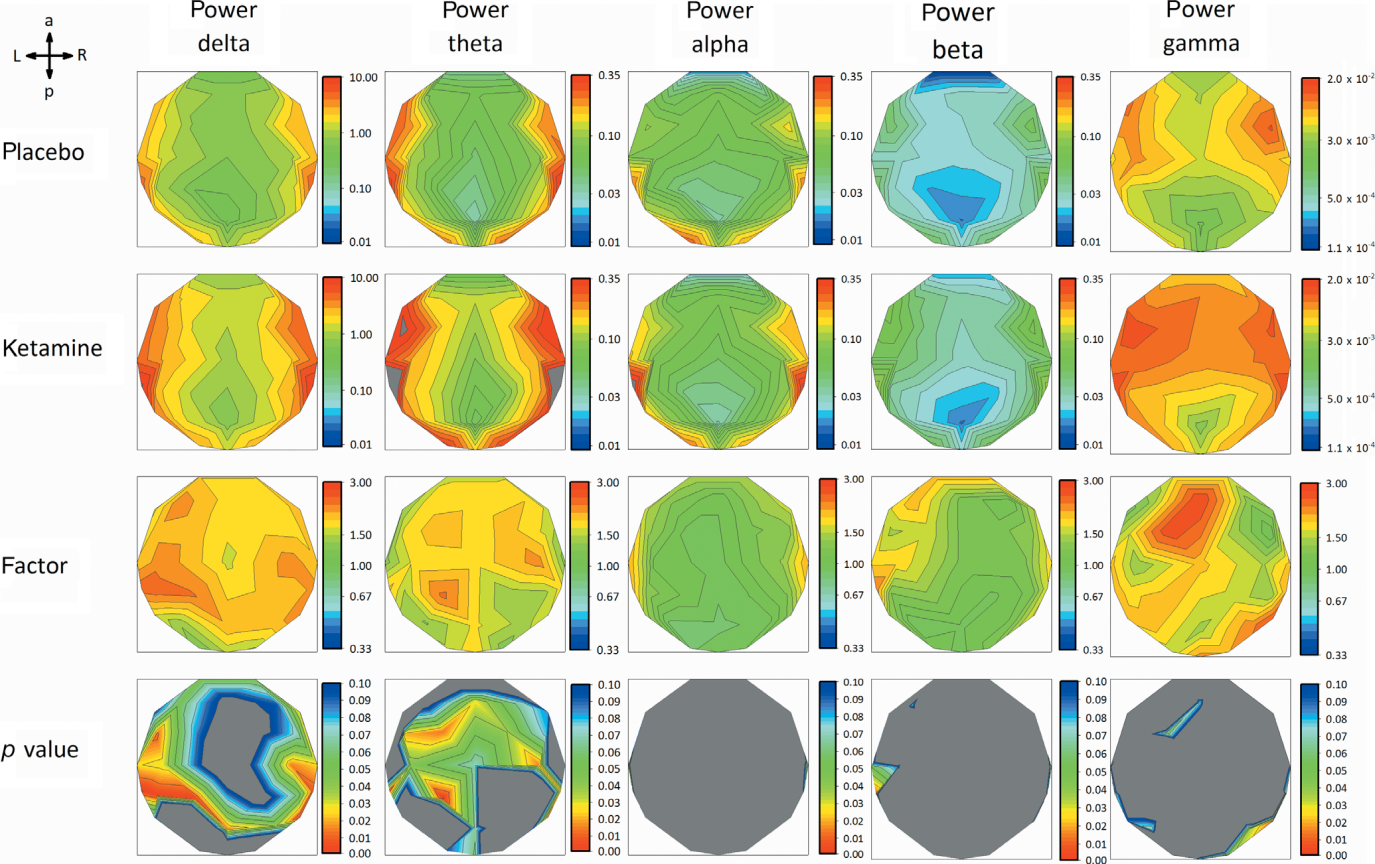
Row 1 and 2 show heatmaps of geometric mean FFT_P_ for all 30 electrodes of placebo and ketamine condition for five frequency bands (delta: 0.53–4 Hz; theta: 4–8 Hz; alpha: 8–12 Hz; beta: 12–25 Hz; gamma: 30–50 Hz). Row 3 shows heatmaps of factors of how the GM FFT_P_ of the two conditions scale to each other (red = higher GM FFT_P_ for ketamine condition, blue = higher GM FFT_P_ for placebo condition, green = equal GM FFT_P_). Please note logarithmic scaling for row 1 to 3. Row 4 shows heatmaps of uncorrected *p* values <.1 (one‐way repeated measure ANOVA, blue *p* < .1, green *p* < .05, red *p* < .001). Despite of some electrodes, in alpha and beta, no clustering of electrodes shows neither high scaling factors, nor significant differences between conditions. In delta and theta, cluster of electrodes with high scaling factors and significantly different (*p* < .05) FFT_P_ over conditions were observable for parietal‐temporal regions and fronto‐central regions, respectively. Highest scaling factors could be seen in fronto‐central electrodes for gamma but without significant difference (*p* < .05) between conditions

Figure 3 shows the GM FFT_P_ for five commonly known frequency bands (delta, theta, total alpha [alpha1 + alpha2], beta, and gamma) as a heatmap of all EEG‐electrodes averaged over subjects. Heatmaps in row one and two plots FFT_P_ for placebo (FFT_P,Pl_) and ketamine (FFT_P,Ke_) conditions, respectively (note different logarithmic scaling for different frequency bands). In row three, heatmaps of the ratio of GM FFT_P_ for ketamine versus placebo condition are plotted. Here, for delta power (Figure 3, column 1), GM FFT_P_ of ketamine condition exceeds GM FFT_P_ of placebo condition by a scaling factor of 2.3 in maximum (electrode CP5) with statistically significant increases of FFT_P,Ke_ mainly for parieto‐temporal electrode positions (*p*_uncorrected_, Figure 3, row 4). For theta power, GM FFT_P_ of ketamine condition exceeds GM FFT_P_ of placebo condition by a scaling factor of 2.3 in maximum (electrode CP1) with statistically significant increases of FFT_P,Ke_ for fronto‐medial electrode positions. Since CP1 is also strongly affected by delta activity, it is not possible to disentangle the overlapping impact of each frequency band. Therefore, we focused our subsequent regression analyses of theta power (see below) on the electrode with next smaller scaling factor (C4, factor of 2.1) but significant increase of FFT_P,Ke_ exclusively for theta. For gamma, several electrodes were close to reach statistical significance (*p*_uncorrected_ < .1). Even so, especially frontal electrode positions (Fz) showed the highest scaling factor (2.7) of our analyses across frequency bands. Distant from the frontally pronounced numerical increase of gamma activity with high scaling factor, statistical significance was reached at a right parietal electrode (P4). Apart from a single electrode in the left temporal region with a statistically significant difference of beta FFT_P_, statistically significant differences for alpha and beta FFT_P_ were not seen. Furthermore, we observed an energy shift of FFT_P_ to slower frequencies within the alpha frequency band in the ketamine condition (see Figure S4).

**Figure 4:**
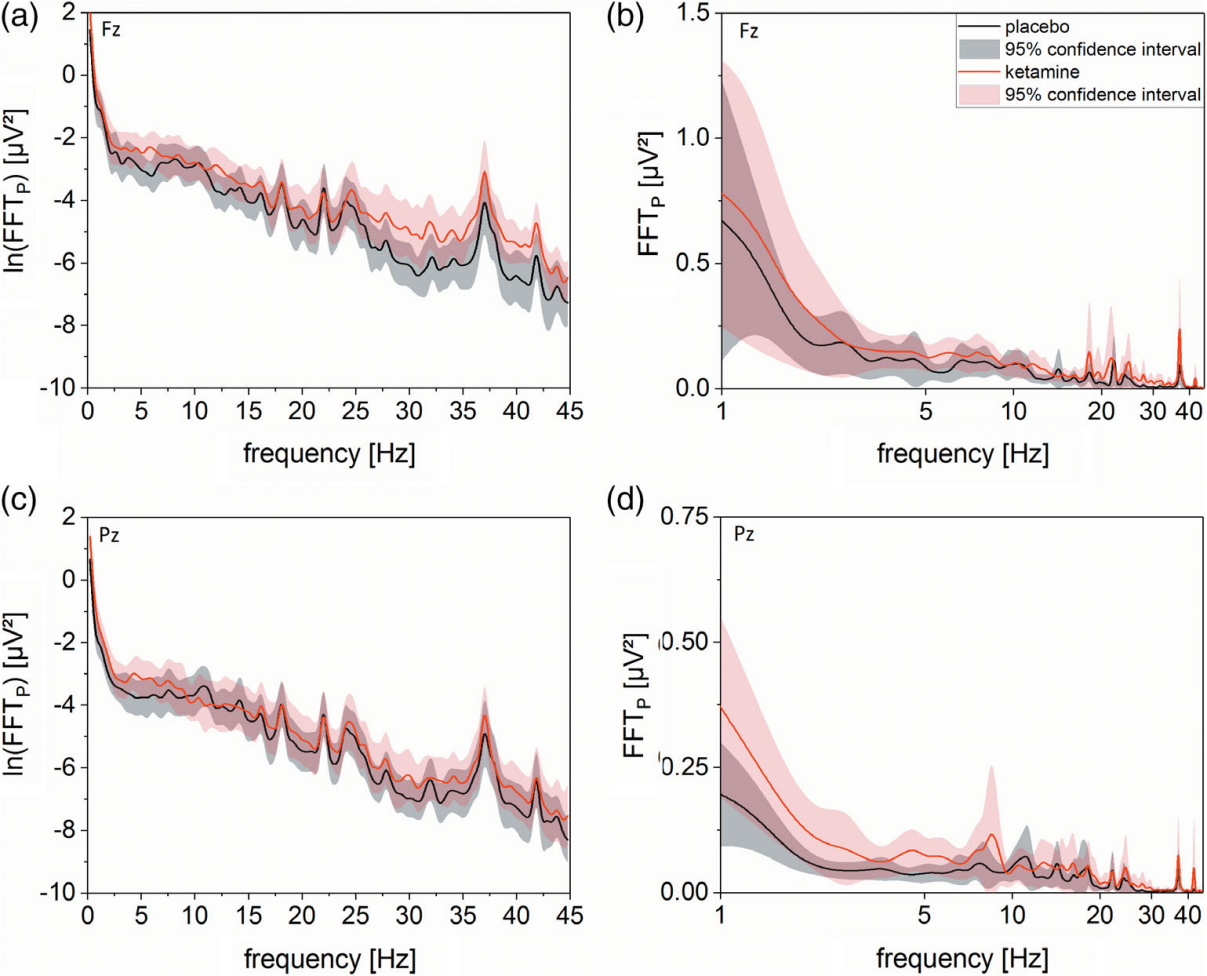
Left side shows b‐spline smoothed mean logarithmic FFT_P_ of placebo (black) and ketamine (red) condition of electrode Fz (a) and Pz (b) against frequency bins from 0 to 45 Hz (bin‐size = 0.48) with regarding 95% confidence interval (light black and light red, respectively). For both electrodes, overlapping graphs could be seen for frequency ranges from 8 to 25 Hz indicating no ketamine related differences. For Fz electrode, the graphs start to spread open at frequencies higher 25 Hz with higher ln(FFT_P_) for ketamine condition. Same could be seen for both electrodes for frequencies between 1 and 7 Hz. Please be aware that for both electrodes, the energy content of small frequencies is at least one magnitude higher than those from high frequencies. For illustrative reasons and to account for the higher energy content of small frequencies, we plotted on the right side of (a) and (b) for the corresponding electrodes the mean FFT_P_ of both conditions against a logarithmically scaled frequency axis. This leads to an emphasis on the difference between ketamine and placebo conditions at lower frequencies

In Figure 4, the group mean spectral power distribution, including 95% confidence intervals, is displayed both for the placebo and ketamine condition. It is shown (a) how absolute EEG power values are distributed in the frontal and parietal region and (b) how the EEG frequency spectrum is shifted in these brain regions between placebo and ketamine. Overall, a shift of energy to slow and fast wave frequencies is seen in the ketamine condition. For a more in‐depth EEG signal analysis of ketamine effects, see Results 1.3–1.5 in Supporting Information.

### 3.3 Ketamine‐induced electric activity changes correlate with corresponding BOLD activation during rest

**Figure 5:**
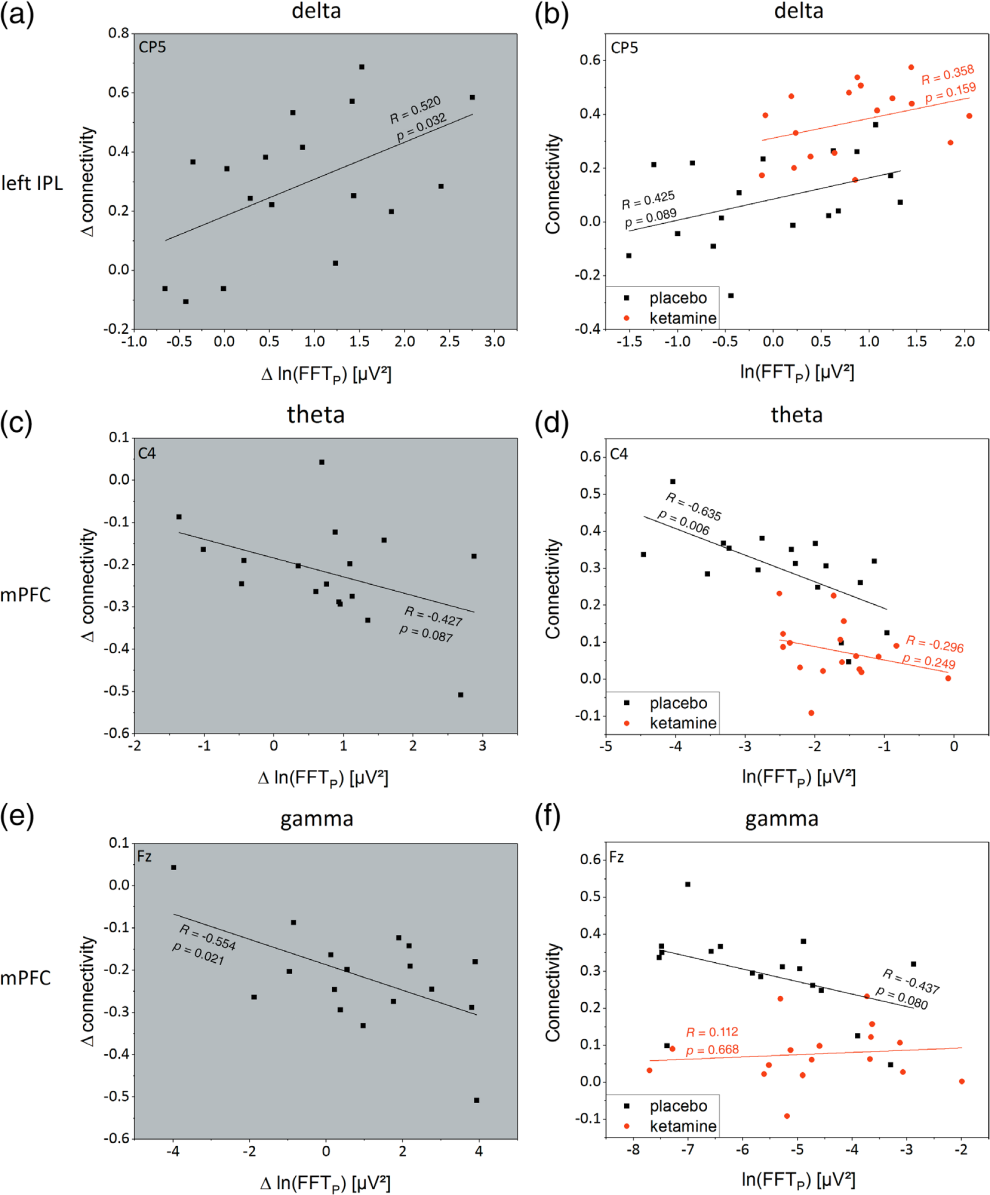
Plots with gray background show the individual difference (ketamine − placebo) of the logarithmic EEG Power (Δln[FFT_P_]) against the matching individual difference of default mode network functional connectivity (Δconnectivity). Plots with white background show the individual ln(FFT_P_) against corresponding functional connectivity for placebo (black) and ketamine condition (red) with associated linear regressions (lines). (a) For delta, an increase in Δln(FFT_P_) of electrode CP5 is significantly accompanied with an increased Δ in left IPL‐PCC/precuneus functional connectivity with a Pearson's *R* of 0.52 (*p* = .032). This is related to the right shifted parallel offset of the linear regression of ketamine condition shown in (b) with higher FFT_P_ and functional connectivity values for ketamine condition. (c, d) A comparable analysis of theta for C4 electrode and DMN functional connectivity between mPFC and PCC/precuneus shows a trend‐wise negative correlation for Δ analysis with a significant decreased correlation for placebo condition (*R* = −0.635, *p* = .006). For ketamine condition, a floor effect with connectivity values close to zero could be observed which leads to low Pearson's *R* of −0.296 (*p* = .249). (e, f) Analyzing ln(FFT_P_) of gamma for electrode Fz against DMN functional connectivity of mPFC, comparable results with a significant negative correlation for Δ analysis and a trend‐wise negative correlation between ln(FFT_P_) and functional connectivity for placebo condition could be seen. Because of the aforementioned floor effect of ketamine related DMN functional connectivity of mPFC, a correlation between ln(FFT_P_) and DMN functional connectivity for ketamine condition could not be observed

Figure 5a,c,e shows regression analyses of the EEG power versus BOLD activation for the individual differences “Δ” (Ketamine minus Placebo) in EEG Power against the corresponding difference of DMN functional connectivity. Figure 5b,d,e shows regression analysis of the EEG power versus BOLD activation for Ketamine and Placebo condition separately. As illustrated in Figure 5a, a significant positive correlation with Pearson's *R* of 0.520 (*p* = .032, *F* = 5.555) was found for the regression analysis of delta power (electrode CP5) against ΔDMN functional connectivity of left IPL. For theta power (electrode C4) against DMN functional connectivity of mPFC, Figure 5c shows a negative trend with Pearson's *R* of −0.427 (*p* = .087, *F* = 3.344). For gamma power (electrode Fz, Figure 5e), a significant negative correlation of ΔEEG power versus Δfunctional connectivity of mPFC could be shown (*p* = .021, *F* = 6.655, *R* = −0.554). When regarding both drug conditions separately, linear regressions in Figure 5b show nonsignificant trends (*p*_Pl_ = .089, *F*_Pl_ = 3.307; *p*_Ke_ = .159, *F*_Ke_ = 2.199) with positive correlations (Pearson's *R*_Pl_ = 0.425 and *R*_Ke_ = 0.358) for delta power versus DMN activity (left IPL). Whereas Figure 5d shows a significant negative linear correlation with a Pearson's *R* of −0.635 (*p* = .006, *F* = 10.131) only for the placebo condition. In the ketamine condition, a floor effect is found with no significant correlation (*R* = −0.296, *p* = .249, *F* = 1.440). In Figure 5e, linear regressions show statistical trends or no significance when comparing EEG power with functional connectivity (*p*_Pl_ = .08, *F*_Pl_ = 3.537, *R*_Pl_ = −0.437, *p*_Ke_ = .668, *F*_Ke_ = 0.191, *R*_Ke_ = 0.112).

## 4 DISCUSSION

This is the first simultaneous rsfMRI/EEG study to assess the effects of ketamine. In this randomized, double‐blind, placebo‐controlled, crossover ketamine trial of 17 healthy Caucasian men, we observed that subanesthetic doses of the noncompetitive N‐Methyl‐D‐Aspartat (NMDA) receptor antagonist S‐Ketamine induces changes of vigilance as assessed by EEG. As hypothesized, we found ketamine effects comparable to what is seen during light sleep with a global shift of energy to slow (and fast) wave EEG frequencies across most parts of the scalp. In addition, we found decreased functional connectivity for frontal parts of the DMN as previously described by Scheidegger et al. (2012) but also an increase of functional connectivity in the parietal brain region. Most importantly, both functional connectivity changes were correlated with EEG after ketamine administration. Overall, our findings suggest that subanesthetic ketamine effects on rsfMRI are related to the ketamine effect on vigilance. However, this ketamine effect on vigilance comes together with opposite effects on cortical network synchronization as indicated by the ketamine effects on rsfMRI functional connectivity. Thus, our findings are compatible with the notion that hyperarousal together with increased functional connectivity during clinical depression is counteracted by the antidepressant compound ketamine through its diminishing effect on EEG vigilance and associated functional connectivity in the PFC. DMN functional connectivity in the parietal cortex using PCC as the seed region was hardly studied in depression (Kaiser, Andrews‐Hanna, Wager, & Pizzagalli, 2015). However, a very recent study of Evans et al. (2018) indicates that depressed patients may show decreased DMN functional connectivity in various brain regions, including parietal regions.

The findings obtained in this study are of quite some interest with regard to recent work highlighting the association of the antidepressant effect of ketamine, vigilance, and neuroplasticity. Duncan et al. (2014) reported that the effect of ketamine on depressive symptoms in treatment‐responsive patients is accompanied by increased slow wave EEG activity during night sleep (increased slow wave activity during sleep is regarded as a marker for sleep depth according to Rechtschaffen & Kales, 1968). Duncan et al. (2014) further reported proportionally increased plasma levels of BDNF (peripheral marker of plasticity) and increases of EEG slow wave activity. The authors discussed these findings in the context of comparable findings in earlier studies in rats (Feinberg & Campbell, 1993) as well as rat and human studies (Esser, Hill, & Tononi, 2007; Riedner et al., 2007; Vyazovskiy, Cirelli, Pfister‐Genskow, Faraguna, & Tononi, 2008) which established that EEG slow wave activity during sleep is a surrogate marker of central synaptic plasticity (synaptic strength and network synchronization). The results of our simultaneous rsfMRI/EEG study indicate that this shift of vigilance with increased slow wave activity together with network synchronization can be observed already during ketamine infusion: slow wave power increases which is paralleled by network synchronization (increased functional connectivity in the parietal cortex). However, as outlined above, this positive correlation between slow wave activity and functional connectivity is only seen in the parietal cortex while a negative correlation is observed in the frontal cortex. In this context, it is important to point out that recent simultaneous EEG/PET and EEG/fMRI‐ASL studies during sleep made observations which are reminiscent of what we found in our ketamine study (Tüshaus et al., 2017). During sleep, they reported about a negative correlation in the PFC between (decreased) blood flow and (increased) slow wave EEG activity whereas in the posterior parts of the brain, a positive correlation with increased blood flow and increased slow wave EEG activity was found. Accordingly, it is conceivable that both during ketamine application and sleep corresponding neuroplastic changes occur in the brain involving deactivation of the PFC and activation of parietal (and occipital) association cortices.

### 4.1 Ketamine affects slow wave EEG power and DMN functional connectivity

In our study, we could show increased slow wave delta and theta activity and a marginal increase of fast wave gamma activity during ketamine administration. Overall, this is in line with recently reported findings of Muthukumaraswamy et al. (2015) who observed comparable changes in frequency content after ketamine administration, with increases in frontal theta (slow wave) and overall gamma activity (fast wave). This seemingly contradicting findings of increased slow wave activity on one hand and fast wave activity on the other hand is best explained in the context of ketamine effects on vigilance regulation, that is, increased slow and fast wave frequency activity is seen during sleep stage 1—a dynamic transition state between wake and sleep which is characterized by CAPs (Parrino et al., 2014). Since sleep stage 1 is unstable by definition, this may also in part explain why different studies in the past reported nonidentical subanesthetic ketamine effects on the EEG frequency spectrum. By extension, one would expect that any observed subanesthetic ketamine effects strongly depend on the so‐called boundary conditions. For instance, in contrast to some other studies (de la Salle et al., 2016; Rivolta et al., 2015), we observed only small ketamine effects on EEG gamma activity in our study. One possible explanation is certainly methodological differences, that is, simultaneous fMRI/EEG data acquisition in our study as opposed to unimodal EEG or MEG in those earlier studies (see Result 1.2 in Supporting Information). However, other possible explanations exist as well. Thus, Rivolta et al. (2015) used an initial bolus for ketamine administration (10 mg) which would be a 25% higher dose than what we used. In addition, in the study of Rivolta and colleagues, the ketamine dose for continuous infusion differs. They administered a dose less than half of the one we used (0.006 mg kg^−1^ min^−1^ vs. 0.015625 mg kg^−1^ min^−1^). Another difference was that the resting‐state measurement was started 10 min later than in our study (45 vs. 35 min after initial bolus injection) and their measurement lasted only 4 min (vs. 8.5 min in our study). Comparable considerations have to be applied when comparing our study with the study of de la Salle et al. (2016). Of note, different from our study, they also used racemic ketamine (S‐Ketamine/R‐Ketamine = 50:50), which differ in its metabolic effects, such that S‐Ketamine affects more circumscribed brain regions with high NMDA receptor densities (Vollenweider, Leenders, Oye, Hell, & Angst, 1997) and thus local circuit gamma band oscillations might be preferentially affected. This kind of different boundary conditions may also play a role when looking at molecular effects of subanesthetic ketamine, for example, cortical glutamate concentrations. The postulated net positive effect of subanesthetic ketamine on excitatory transmission by inducing excessive release of glutamate (Kim et al., 2011; Rowland et al., 2005; Stone et al., 2012) may thus appear in a different light, that is, a more or less transient increase of glutamate release may rapidly be counteracted by opposing molecular mechanisms during subanesthetic ketamine administration depending on the specific boundary conditions.

In the present study, we showed decreased functional connectivity for frontal parts of the DMN. This is in line with recently published rsfMRI studies on the effects of (sub‐)anesthetic ketamine on human brain function. The study of Scheidegger et al. (2012) and Bonhomme et al. (2016) described decreased prefrontal DMN functional connectivity after ketamine administration. This ketamine‐induced decrease of functional connectivity appears to normalize transiently 1 hr after drug application, decreasing again after 24 hr, which in turn is associated with higher Gln/Glu changes (Li et al., 2018). Interestingly, Bonhomme et al. (2016) found a significant negative linear relationship between the depth of sedation (with increasing ketamine doses) and mPFC DMN connectivity. In this context, a study of Purdon et al. (2013) needs to be mentioned showing that with increasing depth of anesthesia, the power of low frequency EEG power is increasing in the frontal brain region. This again is in line with our observations suggesting a relationship between decreased functional connectivity and a reduced level of vigilance following ketamine administration.

### 4.2 Slow wave EEG power and network connectivity are related

To our knowledge, we could show for the first time a negative relationship between frontal DMN functional connectivity and frontal theta power. The negative correlation of frontal DMN functional connectivity with frontal theta (and gamma) power for placebo condition gets lost for ketamine condition (see Figure 5). This is of some interest with regard to pharmacodynamic measurements of ketamine effects (or related drug compounds). The decrease of prefrontal functional connectivity after ketamine administration results in a floor effect of the functional connectivity, which means that the ketamine related effect levels out because functional connectivities are close to zero. Accordingly, one would prefer EEG rather than fMRI measurements when it is the aim to measure ketamine effects. On the other hand, we also could show a positive relationship of bilateral IPL connectivity to PCC and slow wave delta activity. Here, a less restricted range of values is seen for parietal functional connectivities. In any case, for pharmacodynamic studies, it appears preferable to use both EEG and fMRI (ideally with simultaneous data acquisition). In some cases, EEG is the more sensitive tool with a wide range of measurement values. In fact, EEG‐informed fMRI analysis may even help to improve the sensitivity of fMRI to detect drug effects as recently demonstrated by us for nicotinic compounds (Warbrick et al., 2012). In addition, using both data sets together (EEG and fMRI) may help to build confidence in any observed drug effect when a consistent picture is emerging across modalities—most notably in small studies with limited statistical power such as in clinical Phase‐I or Phase‐II studies.

As a limitation of this study, one has to mention, that our study sample—as typical in the framework of a clinical Phase‐0/Phase‐I study (proof‐of‐concept)—only included healthy young men. Thus, the relevance of the bolus‐infusion protocol to antidepressant action could not be investigated and might be of interest for future studies. Furthermore, ketamine affects other physiological parameters like heart rate variability or blood pressure, too. For example, Komatsu et al. (1995) showed that ketamine reduces heart rate variability. However, recently Chang et al. (2013) showed that heart rate variability is associated with functional connectivity of particular brain regions. This is accompanied by a study of Jennings, Sheu, Kuan, Manuck, and Gianaros (2016) who could show that a reduced heart rate variability is correlated with reduced DMN connectivity of mPFC. Thus, based on the known association of vigilance reduction and decreased heart rate variability (Penzel, Kantelhardt, Lo, Voigt, & Vogelmeier, 2003), these results support our findings that ketamine effects on vigilance can be measured with simultaneously measured fMRI/EEG. Another difficulty in rsfMRI measurements with continuous ketamine infusion is the fact that with longer measurement time, subjects tend to move or start to feel unwell. Both might be related to the lack of a task and an individual increased focus on the inner self and one's own emotional state (Andrews‐Hanna, 2012) after ketamine administration. We have accounted for this by reducing our analysis on the first part of our 20 min lasting resting‐state experiment, although a comparison analysis of first and second half experimental data shows no reliable difference.

## 5 CONCLUSION

We could show a relationship of altered EEG activity with DMN connectivity changes consistent with the notion of a ketamine‐induced state of decreased vigilance (sleep stage 1 like). Our findings can be used as a mechanistic neurophysiological model to explain the antidepressant action of ketamine and related drug compounds. By extension, this neurophysiological model based on simultaneous fMRI/EEG may also provide a heuristic framework to improve our understanding of the effects of ketamine and related drugs on brain function in comparable pharmacofMRI studies (Mehta et al., 2018).

## CONFLICT OF INTEREST

G.W. is president of Pharmaimage Biomarker Solutions, Inc. (Boston, USA) and CEO of Pharmaimage Biomarker Solutions GmbH (Berlin, Germany): http://www.pi-pharmaimage.com. P.d.B. and M.L. are employees at Janssen Pharmaceutica/Janssen‐Cilag. N.Z. is holding a part‐time position at Pharmaimage Biomarker Solutions GmbH. The other authors declare no conflict of interest.

## Supporting information


**Appendix S1:** Supplementary Results

## Data Availability

The data that support the findings of this study are available from the corresponding author upon reasonable request.
