# Supplementary material for: Ketamine effects on default mode network activity and vigilance: A randomized, placebo‐controlled crossover simultaneous fMRI/EEG study
Source: Hum Brain Mapp. 2019 Sep 18;41(1):107–19. doi: 10.1002/hbm.24791 (PMC7268043; doi:10.1002/hbm.24791)
Supplement: Supplementary file 1 — Appendix S1: Supplementary Results [file HBM-41-107-s001.pdf]

Supplementary Results

1.1. Seed based network connectivity patterns in the placebo and ketamine condition

For seed based analysis of the default mode network connectivity, the PCC/precuneus area is used as a seed of interest. Performing a F-test for second level statistical analysis, a typical clustering of significant activation patterns is observed. The cluster covers areas including the medial prefrontal cortex, as well as left and right hemispheric parietal lobes and PCC/precuneus areas. Different connectivity patterns for the placebo and ketamine condition (Figure S1, two-sided F-statistic height threshold T>8.5, FWE corrected cluster threshold p<0.01) is observed, and shown in the main text body (result section, Figure 2). A detailed description of the significantly different activated brain regions for the contrast ketamine > placebo is presented in Table S1 (FWE corrected p=0.05).

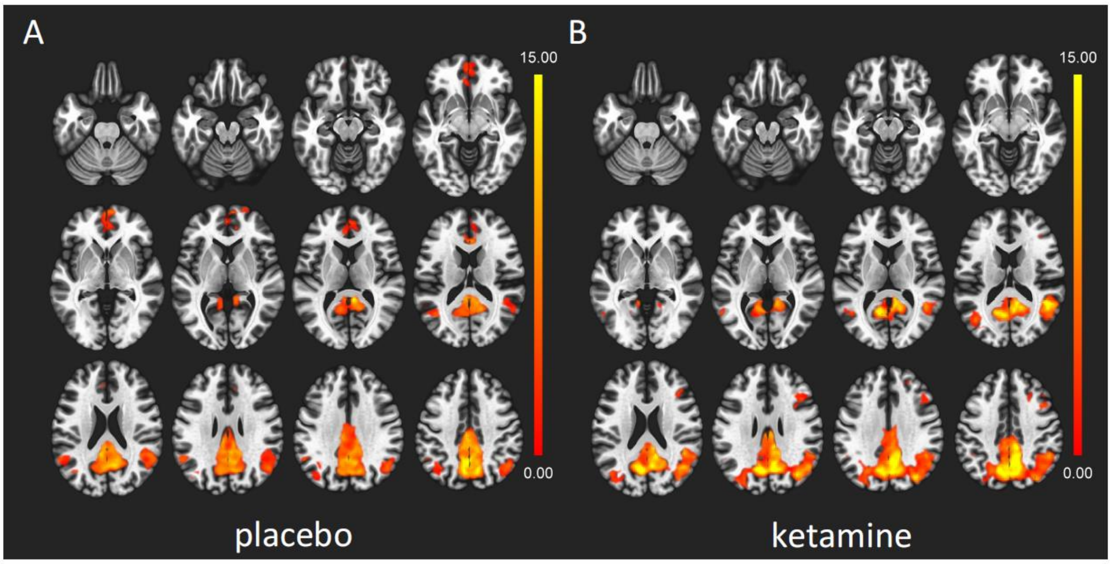

Figure S1 Activations shown are the intersections (axial, 6mm intersection distance) of the DMN z-transformed network maps of the placebo (a) and ketamine (b) condition (color coded T-values). Note the decrease of frontal connectivity and the increase of parietal connectivity to the PCC/precuneus (seed) area for ketamine condition.

1.2 Gamma band activity measured with simultaneous EEG/MRI setup

It is important to mention that gamma band activation of simultaneously measured EEG/fMRI data is contaminated by technical artifacts within a frequency range >30Hz. Whereas the gradient artifact which is stable over time can be sufficiently removed by template subtraction methods, and the Helium-Pump artifact can be avoided by simply switching off the Helium-pump like in our study, other artifacts like cardio ballistic artifacts (CBA) or artifacts from the internal ventilation system (4) are not that easy to handle. Currently developed technical solutions like simultaneous measurements with carbon wired loops (5) or multiband fMRI

techniques (6), which would help to clean the data from such artifacts, were not developed at the time when we conducted the EEG/fMRI measurements. As common post processing algorithms cannot sufficiently clean the data from such artifacts (5), it is difficult to interpret differences in gamma band activity in simultaneously measured EEG/fMRI data. Therefore we decided to use filter settings with a relatively sharp cutoff at 45 Hz with a slope of 8db. This focused our analysis to frequencies lower than 50Hz since with our settings at less than 50 % of energy of frequencies > 50Hz remain (Figure S2). Nevertheless, this affects both conditions equally and so it could not explain the differences we had found between conditions.

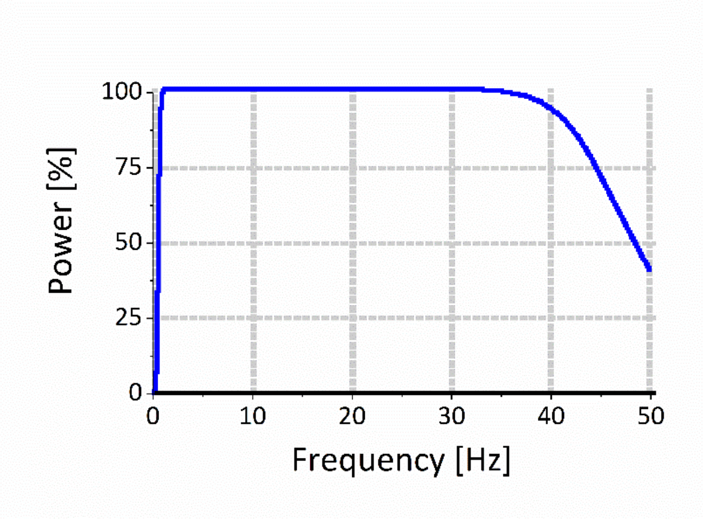

Figure S2 shows the remaining amount of EEG Power in percent after applied filter settings (zero phase shift Butterworth filters: order 4 low cutoff filter of 0.53Hz , order 8 high cutoff filter of 45Hz). Note that less than 50 % of EEG power for frequencies > 50Hz remain.

1.3 Spectral edge frequency (SEF)

Commonly, SEFs from frontal electrodes are used in anesthesiology, to assess the depth of anesthesia. SEF is defined as the frequency below which a certain amount of total power is concentrated (7, 8). For our analysis we use an amount of 90% of total power. SEF analysis was conducted for four different brain regions covering the head surface which were defined by electrode positions (frontal: FP1, FP2, F3, F4, F7, F8; temporo-central left hemisphere: T7, FC1, FC5, C3, CP1, CP; temporo-central right hemisphere: T8, FC2, FC6, C4, CP2, CP6; parieto-occipital: TP9, TP10, P7, P8, P3, P4, Pz, O1, O2, Oz). The resulting individual mean SEFs were used for statistical analysis.

Figure S3 show the mean SEF and SD for placebo (black) and ketamine (red) condition of frontal, parieto-occipital, temporo-central left and right brain regions. Two-Way Repeated Measure ANOVA gave significant differences in SEF with lower SEF for ketamine condition (SEF_K, $p < 0.017$) in frontal brain regions and higher SEF_K for parieto-occipital brain regions ($p < 0.02$). Temporo-central regions show no significant differences in SEF between drug conditions. It is well known that the EEG spectral edge frequency (SEF), measured with electrodes attached at frontal head positions, is decreasing with increasing anaesthesia and it is nowadays used as clinical marker for the depth of anaesthesia. Here we could show that such decrease in SEF is also true when applying ketamine in subanaesthetic doses. Furthermore, we found an increase in SEF for parietal-occipital brain regions, which normally is not detectable with standard clinical setups. One may consider this observation, when

frontal electrode positions are not applicable for clinical monitoring and thus, when there is a need for using other electrode positions.

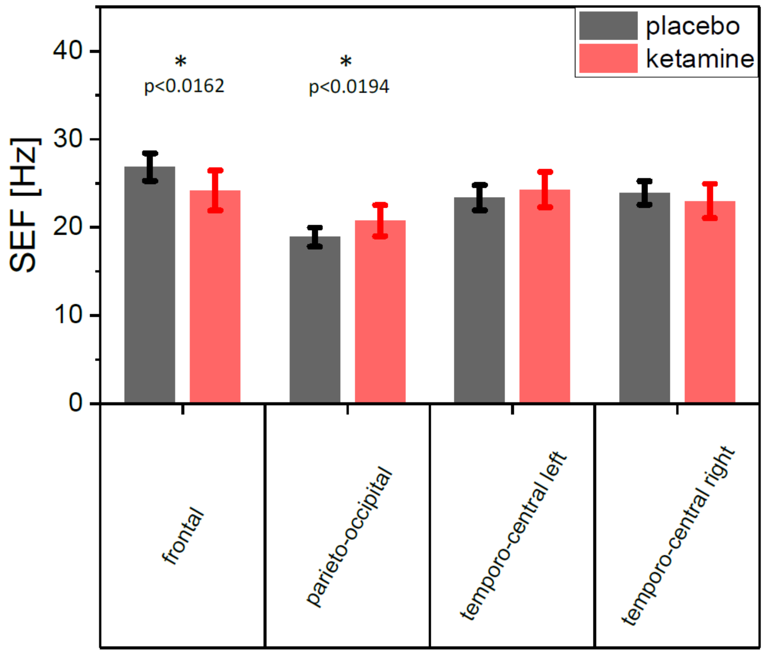

Figure S3 mean SEF±SD for four different brain regions for placebo (black) and ketamine condition (red). Underlying data are SEFs of neighbouring electrode positions for frontal, parieto-occipital, temporo-central left and temporo-central right head surface areas based on the International 10-20 system. Significant differences between conditions could be shown only for frontal ($p < 0.017$) and parieto-occipital brain regions ($p < 0.02$), with lower and higher mean SEF for ketamine condition, respectively.

1.4 Energy shifts from alpha2 to alpha1

Purdon et al. (9) showed increased alpha power for frontal brain regions with increasing depth of anaesthesia. Although there are no differences between conditions in alpha FFT_P, for ketamine condition an energy shift of FFT_P to slower frequencies in between alpha is observable. Figure S4 (right part) shows a scatterplot of the geometric mean (GM) FFT_P of alpha1 (red squares) or alpha2 (blue circles) plotted against the GM FFT_P of alpha for placebo (open symbols) and ketamine (closed symbols) condition. The $FFT_{P,\,GM}$ data of all electrodes show no differences in the relationship of alpha1 to alpha (a1/a) and alpha 2 to alpha (a2/a) for placebo condition (Wilcoxon Signed Rank Test with paired samples, $p = 0.81$, $Z = -0.25$) but a statistically significant different distribution of the ratios a1/a and a2/a for ketamine condition (Wilcoxon signed rank Test with paired samples, $p << 0.001$, $Z=-4.71$). This is supported when performing linear model fitting of the data (Pearson´s $R > 0.99$) with comparable slopes m close to 1 ($m_{a1/a} = 0.95±0.02$, $m_{a2/a} = 0.98±0.02$) for placebo condition and different slopes $m_{a1/a} =1.09±0.02$ and $m_{a2/a} = 0.85±0.02$ for ketamine condition. For better visualization of the head distribution of the aforementioned energy shift, the left part of Figure 3 shows heatmaps of FFT_P ratios “alpha1 vs. alpha” and “alpha2 vs. alpha” for placebo and ketamine condition. An energy shift to alpha 1, namely increasing ratios > 1 for “alpha1 vs. alpha” and decreasing ratios < 1 for “alpha2 vs. alpha”, for ketamine condition in comparison

to placebo condition could be seen, with mainly frontal, temporal and parietal regions involved.

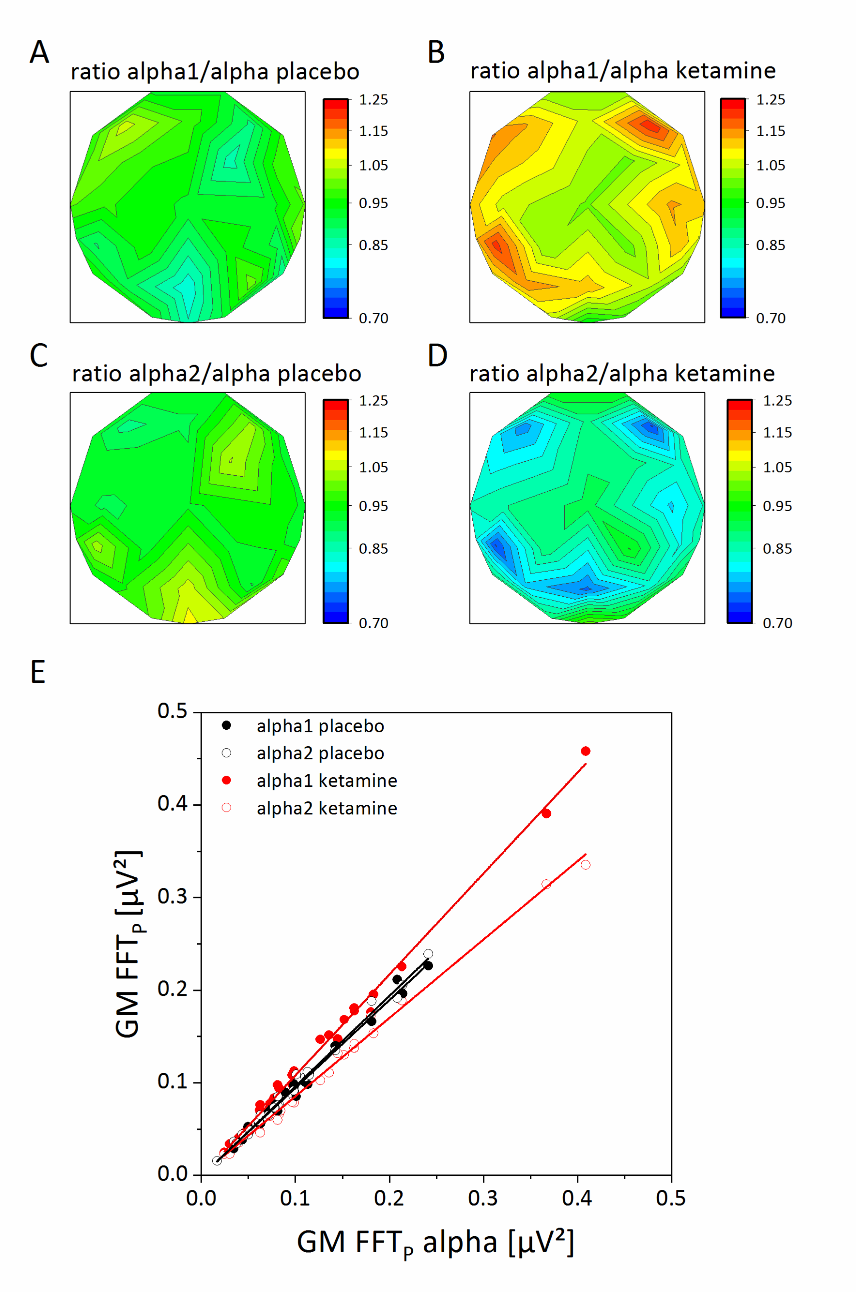

Figure S4 a-d) show heatmaps of FFT_P ratios "alpha1 vs. alpha" **(a-b)** and "alpha2 vs. alpha" **(c-d)** for placebo **(a, c)** and ketamine **(b, d)** condition. **e)** Shows a scatterplot of GM FFT_P of alpha1 (closed symbols) or alpha2 (open symbols) plotted against the GM FFT_P of alpha for placebo (black) and ketamine (red) condition (lines were corresponding line fits).

Of further interest, this increase in alpha1 power for ketamine condition is accompanied by increased fronto-parietal coherence of the right hemisphere, which cannot be equated with increased functional connectivity between anterior and posterior brain structures.

1.5 Coherence analysis

For analyzing the magnitude squared coherence between different electrodes, we used the power spectra of 15 electrode pairs of interest that are representative of connections within or between both hemispheres (intrahemispheric left hemisphere: F3C3, F3P3, F3T7, C3P3, C3T7, P3T7; intrahemispheric right hemisphere: F4C4, F4P4, F4T8, C4P4, C4T8, P4T8; interhemispheric: F3F4, C3C4, P3P4).

alpha1 coherence analysis: Figure S5 plots the magnitude squared coherence between two electrodes as mean coherence values between 0 (low connectivity) and 1 (high connectivity) + SD of alpha1 FFT_P for placebo (black) and ketamine (red) condition. The plotted results were divided into interhemispheric and intrahemispheric electrode pairs. As shown in Figure S5 ketamine leads to higher coherence values compared to placebo condition for right hemispheric electrode pairs. Similar effects for left hemispheric pairs could not be shown. Additionally statistical significant differences between conditions could be shown for the central interhemispheric electrode pair (C3-C4, $p = 0.025$, $F = 6.12$), but we have to mention the very small coherence values below 0.134. The other frequency bands show nearly no significant differences between conditions.

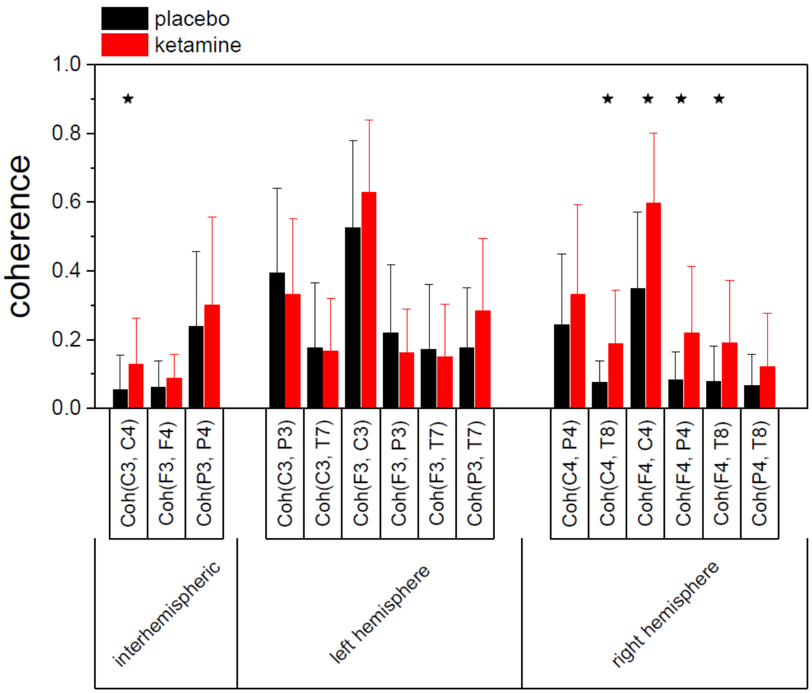

Figure S5 Coherence analysis for inter- and intrahemispheric electrode pairs color-coded for conditions (placebo = black, ketamine = red). Bars + tickmarks represent mean + SD coherence between 0 (low connectivity) and 1 (high connectivity) for alpha1 FFT_P. One-way repeated measure ANOVA gave significant differences ($p<0.05$) mainly for right hemispheric electrode pairs with higher coherences of ketamine condition.

**mPFC**	240 voxels	9% of Cingulate Gyrus, anterior division
	**212 voxels**	**16% of Paracingulate Gyrus Left**
	**190 voxels**	**19% of Frontal Medial Cortex**
	157 voxels	14% of Subcallosal Cortex
	156 voxels	11% of Paracingulate Gyrus Right
	45 voxels	1% of Frontal Pole Left
	176 voxels	not-labeled
**left IPL**	**393 voxels**	**27% of Superior Parietal Lobule Left**
	321 voxels	6% of Lateral Occipital Cortex, superior division Left
	**240 voxels**	**25% of Supramarginal Gyrus, anterior division Left**
	105 voxels	3% of Postcentral Gyrus Left
	200 voxels	not-labeled
**right IPL**	**219 voxels**	**15% of Superior Parietal Lobule Right**
	72 voxels	1% of Lateral Occipital Cortex, superior division Right
	17 voxels	1% of Supramarginal Gyrus, posterior division Right
	11 voxels	Angular Gyrus Right or not labelled

Table S1 shows the amount of FWE corrected significant voxels (two-sided, p = 0.05) for the contrast ketamine > placebo. The size of the related brain area in percent is based on Harvard Oxford Atlas. Brain regions which involve more than 15 % of an atlas region are highlighted in bold.

1. Rivolta D, Heidegger T, Scheller B, Sauer A, Schaum M, Birkner K, *et al.* (2015): Ketamine Dysregulates the Amplitude and Connectivity of High-Frequency Oscillations in Cortical–Subcortical Networks in Humans: Evidence From Resting-State Magnetoencephalography-Recordings. *Schizophr Bull*. 41: 1105–1114.

2. de la Salle S, Choueiry J, Shah D, Bowers H, McIntosh J, Ilivitsky V, Knott V (2016): Effects of Ketamine on Resting-State EEG Activity and Their Relationship to Perceptual/Dissociative Symptoms in Healthy Humans. *Front Pharmacol*. 7: 348.

3. Vollenweider FX, Leenders KL, Oye I, Hell D, Angst J (1997): Differential psychopathology and patterns of cerebral glucose utilisation produced by (S)- and (R)-Ketamine in healthy volunteers using positron emission tomography (PET). *Eur Neuropsychopharmacol*. 7: 25–38.

4. Nierhaus T, Gundlach C, Goltz D, Thiel SD, Pleger B, Villringer A (2013): Internal ventilation system of MR scanners induces specific EEG artifact during simultaneous EEG-fMRI. *Neuroimage*. 74: 70–76.

5. van der Meer JN, Pampel A, Van Someren EJW, Ramautar JR, van der Werf YD, Gomez-Herrero G, *et al.* (2016): Carbon-wire loop based artifact correction outperforms post-processing EEG/fMRI corrections-A validation of a real-time simultaneous EEG/fMRI correction method. *Neuroimage*. 125: 880–894.

6. Uji M, Wilson R, Francis ST, Mullinger KJ, Mayhew SD (2018): Exploring the advantages of multiband fMRI with simultaneous EEG to investigate coupling between gamma frequency neural activity and the BOLD response in humans. *Hum Brain Mapp*. 1–15.

7. Levy WJ (1984): Intraoperative EEG patterns: implications for EEG monitoring. *Anesthesiology*. 60: 430–4.

8. Schwender D, Daunderer M, Mulzer S, Klasing S, Finsterer U, Peter K (1996): Spectral edge frequency of the electroencephalogram to monitor “depth” of anaesthesia with isoflurane or propofol. *Br J Anaesth*. 77: 179–84.

9. Purdon PL, Pierce ET, Mukamel EA, Prerau MJ, Walsh JL, Wong KFK, *et al.* (2013): Electroencephalogram signatures of loss and recovery of consciousness from propofol. *Proc Natl Acad Sci U S A*. 110: E1142-51.
